# Polychlorinated biphenyls and depression: cross-sectional and longitudinal investigation of a dopamine-related Neurochemical path in the German HELPcB surveillance program

**DOI:** 10.1186/s12940-017-0316-3

**Published:** 2017-10-10

**Authors:** Petra Maria Gaum, Monika Gube, Thomas Schettgen, Franziska Maria Putschögl, Thomas Kraus, Bruno Fimm, Jessica Lang

**Affiliations:** 1 Institute for Occupational Medicine, University Hospital RWTH Aachen, Pauwelsstraße 30, 52074 Aachen, Germany; 2 Health Office of the city and area of Aachen, Trierer Straße 1, 52070 Aachen, Germany; 3 Department of Psychiatry and Psychotherapy, Central Institute of Mental Health, J 5, 68159 Mannheim, Germany; 4 Clinic for Neurology, University Hospital RWTH Aachen, Pauwelsstraße 30, 52074 Aachen, Germany

**Keywords:** Polychlorinated biphenyls, Neurotoxicity, Neurotransmitter metabolites, Dopamine, Homovanillic acid, Vanillylmandelic acid, Depressive symptoms, Humans, Adults

## Abstract

**Background:**

Exposure to polychlorinated biphenyls (PCBs) is associated with depressive symptomatology. A cause of depressive symptoms is a disturbance in the neurotransmitter system of dopamine (DA). Animal as well as human studies report that PCBs can influence the DA system. This study examined whether PCB-related depressive symptoms are affected by DA metabolites in humans with high PCB body burden.

**Methods:**

This study is part of the German HELPcB surveillance program (Health Effects in high Level exposure to PCB) for occupationally exposed workers and their relatives. Data was collected from 178 participants on two measurement time points (t1 and t2) with a one-year time lag in between the two time points. PCBs were analyzed in plasma via human biomonitoring and a validated questionnaire was used to identify existence and severity of depressive symptoms. As a surrogate for DA, we measured its metabolites homovanillic acid (HVA) and vanillylmandelic acid (VMA) in urine. Mediation analyses were performed to test whether the association between PCB exposure and severity of depressive symptoms is mediated by urinary concentration of DA metabolites HVA and VMA. The mediation was tested with the SPSS macro MEDIATE.

**Results:**

We found a significant mediation over time for lower-chlorinated, higher-chlorinated and dioxin-like PCBs. The positive association between PCB exposure with severity of depressive symptoms was mediated by the main DA metabolite HVA. At t1 a higher exposure with PCBs was associated with lower concentration in urinary HVA. A reduced HVA concentration at t1 was correlated with increased depressive symptoms severity at t2. No meditations were found for VMA.

**Conclusions:**

This work indicates that the association of PCB exposure and an increase of depressive symptoms after one year is mediated by the DA metabolite HVA as a surrogate for DA. These are first steps towards finding an explanation for an underlying neurochemical pathomechanism of PCB-related depressive symptomatology.

**Electronic supplementary material:**

The online version of this article (10.1186/s12940-017-0316-3) contains supplementary material, which is available to authorized users.

## Background

Polychlorinated biphenyls (PCBs) are a group of chemical substances, which had been commonly used in industrialized nations, because of their chemical and thermic stability, until their worldwide ban by the UNEP (United Nations Environment Programme) in 1995 [1]. PCBs can still be detected in the environment today, because of their high persistence. This high persistence is the reason why the general population has a background burden to PCBs with possible adverse health effects. Today individuals with higher PCB burden get primarily exposed to it in work contexts; for example during the recycling process of old electric devices produced prior to the ban of PCBs [2]. Exposure to PCBs has been associated with several adverse health effects, including: skin disease, abnormalities in liver function [3] and cancer [4]. Furthermore, preliminary results also suggest a potential negative impact on mental health. Peper et al. [5] found a moderate lower well-being in exposed teachers, who worked in a PCB contaminated school building. Seegal et al. [6] however report no correlation between PCBs and trait anxiety and depressive symptoms in former capacitor workers. In contrast, Kilburn et al. [7] found higher rates of depressive symptoms in PCB-exposed firefighters compared with a control group, but they found no direct association with PCB body burden. Conversely, Fitzgerald et al. [8] report a strong positive correlation between PCB body burden and depressive symptoms in elderly PCB exposed Hudson River residents. Similarly, in a prior longitudinal study, we found a higher risk for depressive syndrome in higher PCB-exposed individuals over a period of three years [9]. In summary, findings in the literature indicate a potential association of PCB exposure with depressive symptoms. The aim of this study is to explore potential neurophysiological mechanisms that link individual depressive symptoms to PCB exposure. Many mental disorders are associated with reduced concentrations in neurotransmitters. Especially in depression, lower levels of the monoamine neurotransmitters dopamine, serotonin and norepinephrine can be found [10]. For instance, a low dopamine (DA) level is associated with different types of depression [11] and depressive symptoms (i.e. motor-retardation; [12]). In this study, we focus on the neurotransmitter system of DA as possible path to explain the association of PCB exposure and depressive symptoms.

In the central nervous system (CNS), DA is produced in the presynaptic terminal and discharged from the vesicles into the synaptic cleft. It activates the DA receptors on the postsynaptic terminal and triggers an action potential. Thereafter, the DA transporter (DAT) takes the most of DA back into the presynaptic terminal, whereas another portion is metabolized into homovanillic acid (HVA) and vanillylmandelic acid (VMA) in the synaptic cleft. An alteration in the DA system may result in motor-retardation, a symptom that can also be seen in depressed patients [13]. Furthermore, clinically diagnosed depression is associated with lower levels of the metabolites HVA and VMA. Patients with DSM (Diagnostic and Statistical Manual of Mental Disorders) diagnosed depression show a lower HVA level in blood plasma than healthy controls [14]. Patients with clinically relevant depression have also been found to show a reduced VMA level in the cerebrospinal fluid (CSF) [15], and depressed patients at risk for suicide were found to demonstrate reduced HVA levels in their urine [16].

Neurotransmitter systems can be very sensitive to external influences, such as environmental toxicants like PCBs. A literature review shows that a significant body of research has already investigated the neurotoxic effects of PCBs in animals, and to a much lower extent in humans [17]. The majority of the mentioned studies in the review however examine neurodevelopmental effects, and research in adult humans with occupational exposure to PCBs is still rare until today. To the authors knowledge, Seegal et al. [18] and Putschögl et al. [19] are the only studies that investigate DA-related outcomes in occupational settings. Seegal et al. [18] found that an increase of PCB body burden is associated with a reduced DAT density, but this was only observed in women. Putschögl et al. [19] found a negative association between PCB body burden and urinary HVA as well as urinary VMA after work-related PCB exposure.

**Fig. 1:**
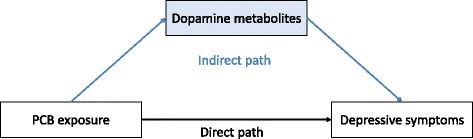
Hypothesized mediation model with direct and indirect path

In this study, we examine whether DA level – reflected by urinary metabolite concentration – mediate the positive association of PCB body burden to depressive symptoms in individuals exposed to PCBs through their occupation (see Fig. 1). In the first hypothesis, we tested the direct path between PCB exposure and depressive symptoms. As reported in previous literature, we expect a positive association. In the mediation hypothesis we measure the urinary concentrations of the DA metabolites HVA and VMA as indicators for central DA level and hypothesize that there is an indirect path between the PCB body burden and depressive symptoms via alterations in the neurotransmitter DA reflected by urinary HVA and VMA level. Related to the indirect effect, we specifically postulate a negative association between PCB body burden and the DA metabolites HVA and VMA and between the DA metabolites and depressive symptoms. Extended to prior research, we further expect to find a cross-sectional as well as a longitudinal mediation between PCB body burden and depressive symptoms through DA metabolites HVA and VMA.

## Methods

### Study population

**Fig. 2:**
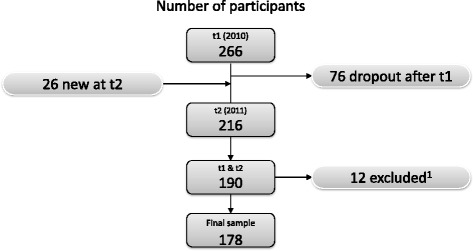
Flow-chart of the number of the study population. Note: t1 = measurement occasion 1, t2 = measurement occasion 2. ^1^excluded due to dopamine relevant medication, such as antidepressants or medication in Parkinson disease

This study was conducted as part of the long-term HELPcB (Health Effects in high Level exposure to PCB) surveillance program for former workers of a recycling company and surrounding companies with occupational PCB exposure and their relatives [20]. All participants received a yearly medical screening as part of the prevention program and the data used in this study was collected from two measurement time points in 2010 (t1) and 2011 (t2). Within these two measurement time points, 292 individuals participated at least at one measurement time (Fig. 2). In comparing drop-outs with the remaining participants, two reasons for a drop-out could be identified [21]. The first reason for remaining in the program was participants' satisfaction with the medical care. Participants who were more dissatisfied with the medical care in the program rather drop-out of the program. The second reason for leaving the program after t1 was related to problems in carrying out everyday tasks due to health problems. Participants who had fewer problems in carrying out their daily tasks rather left the program. A possible explanation may be that participants without or only few problems in carrying out their everyday tasks do not see the need to participate in the program. In total, 178 participants were included in this study, all of which participated in both measurement time points and did not take any dopamine-relevant medication such as antidepressants or Parkinson medication. The included participants did not significantly differ from the excluded ones in age, gender or liver function, nor in the PCB body burden, in depressive symptoms in terms of the sum score of the BDI or in the dopamine metabolites (data not shown). The mean age of the study population was 46.9 years (SD = 12.7 y), of which 155 (87.1%) were men and 23 (12.9%) women. In our study sample five (2.8%) participants left school without a degree, 71 (39.9%) had achieved a secondary school degree, 57 (32.0%) had a junior high school degree and 42 (23.6%) were awarded a university entrance diploma. The education status was unknown for three participants (1.7%).

### Polychlorinated biphenyls

Data collection occurred at a university outpatient clinic for occupational medicine in the morning, where blood samples were collected from each participant for human biomonitoring. Participants were asked not to have breakfast before blood collection. The plasma was analyzed in our laboratory with gas chromatography and electron ionization-mass spectroscopy (please see Schettgen et al. [22, 23] for a detailed description of PCB detection in our study sample). To ensure the reproducibility as a marker for measurement quality of our results the between-day imprecision was determined. For this purpose, a quality control sample was analyzed in each analytical series and the mean values and standard deviation were determined for each congener. The results had a consistency of 5.9% to 7.4% with each other in the considered period indicating a good reproducibility. Furthermore, the accuracy of the determination of the indicator-PCBs in plasma was controlled by biannual successful participation in round robin exercises [24]. Six indicator PCB congeners (28, 52, 101, 138, 153 and 180) and twelve dioxin-like PCBs (77, 81, 105, 114, 118, 123, 126, 156, 157, 167, 169 and 189) were measured as part of the HELPcB program. From the above-mentioned dioxin-like PCBs, the four coplanar PCBs (77, 81, 126 and 169) were excluded from all analyses, as more than 80% of the participants showed values under the limit of detection (LOD). For a similar procedure see Lee et al. [25], Aminov et al. [26] or Fitzgerald et al. [8]. The LOD was 0.01 μg/L plasma and all values under the LOD were divided by two. With the remaining 14 PCB congeners three sum variables were created. According to the number of substituted chlorine atoms and the chemical structure, PCBs were categorized in lower-chlorinated biphenyls (LPCBs), higher-chlorinated biphenyls (HPCBs) and dioxin-like PCBs (dlPCBs). LPCBs have five or less substituted chlorine atoms and are more indicative of occupational exposure or exposure via inhalation, while HPCBs have more than five substituted chlorine atoms and instead reflect nutritional exposure and accumulate in the human adipose tissue over time. DlPCBs consist of lower- and higher-chlorinated PCBs but differ in their dioxin-like chemical structure. Therefore, we generated a third PCB group only with dlPCBs. The lower-chlorinated PCBs (PCB 28, PCB 52, PCB 101) were summed up into the variable LPCBs, the higher-chlorinated PCBs (PCB 138, PCB 153, PCB 180) to the variable HPCBs and the remaining dioxin-like PCBs (PCB 105, PCB 114, PCB 118, PCB 123, PCB 156, PCB 157, PCB 167 and PCB 189) to dlPCBs. For the dlPCBs no WHO toxic equivalency factor (WHO-TEF) was considered, because all used dlPCBs have the same WHO-TEF of 0.00003 [27]. To allow for clearer comparisons between the PCB body burden in our study sample and other study cohorts, we transformed the PCB variable from μg/L plasma in ng/g blood lipid and used the lipid-adjusted variables in all analyses. Cholesterol and triglycerides were detected in the serum and the total lipid level was calculated with the short formula from the CDC (Centers for Disease Control and Prevention [28]: total lipids = (2.27 * total cholesterol) + triglycerides +62.3 mg/dl). Afterwards we divided the PCBs in μg/L plasma by total lipids (g/L serum). The PCB body burden is stable over the measurement occasions. PCB level at t1 is highly correlated with PCB level at t2 (LPCBs: *r* = .96, *p* < .001; HPCBs: *r* = .98, *p* < .001; dlPCBs: *r* = .99; p < .001).

### Transmitter metabolites

Urinary HVA and VMA as the main metabolites of the catecholamines DA are used as indicators to map out the central DA and NE concentration; a higher level of central DA is associated with more urinary metabolites [29, 30]. As a non-invasive method to assess neurotransmitter metabolism and turnover, random urinary samples were collected in the late morning between 9:00 a.m. and 11:00 a.m. The urine samples were stored at −20 °C and the concentration of HVA and VMA were detected via HPLC (high performance liquid chromatography). For a better interpretation of the metabolite concentration in random urine it is necessary to adjust for individual urine density [30, 31]. Therefore, Jaffe color reaction was used to analyze urinary creatinine concentrations and values are expressed as the ratio of HVA to creatinine and VMA to creatinine (HVA/Crea and VMA/Crea; both in μmol/g creatinine). Because the concentration of urinary DA-Metabolites depends to a large extent on liver function, albumin was determined as a marker for liver function in the serum. Furthermore, prior research reports that a higher concentration of urinary DA is associated with post-traumatic stress disorder [32]. In the current study, participants were asked how much they have been bothered by actual and prior traumatic experience.

### Depressive symptoms

Depressive symptoms were measured using the updated version of Beck’s depression inventory (BDI-II; [33]). The BDI-II is a validated self-rating questionnaire that measures the severity of depressive symptoms and consists of 21 items with typical symptoms of depression such as “irritability” or “thoughts about suicide”. The participants were asked to specify how strong their symptoms were in the last two weeks. Each item has four options that range from 0 (the symptom is not manifested) until 3 (the symptom is strongly manifested). The internal consistencies were .91 at t1 and .92 at t2 thus showing a good reliability. To generate the outcome variable for each measurement occasion the answers from all BDI-II-items were added to a sum score. The sum scores can be interpreted as the severity of depressive symptomatology. In the following, we are referring to these outcome variables as “depressive symptoms”. Depressive symptoms at t1 highly correlate with depressive symptoms at t2 (*r* = .79, *p* < 001) and thus they are stable over time.

A statistical description of all relevant variables and their respective reference values are reported in Table 1.

**Table 1:** Sample characteristics in terms of exposure, biochemistry and outcome (*N* = 178)

	reference value	t1			t2		
		Mean ± SD	Median	Range	Mean ± SD	Median	Range
LPCBs^a^	3.9^a**,**c^	402.8 ± 1887.5	21.2	1.3–19,345.0	303.4 ± 1493.2	12.8	1.4–14,090.4
HPCBs^a^	264.8^a**,**c^	964.9 ± 1780.3	331.0	40.5–13,855.3	901.2 ± 1646.7	302.1	43.1–11,794.2
dlPCBs^a^	30.7^a**,**c^	345.4 ± 775.3	62.2	7.0–6051.8	313.7 ± 756.6	53.6	8.3–6342.9
HVA/crea^b^	<42^b**;**d^	19.8 ± 9.2	18.1	6.3–79.0	22.3 ± 12.8	19.2	10.0–115.1
VMA/crea^b^	<30^b**;**d^	15.4 ± 5.7	14.6	7.0–46.0	15.8 ± 6.2	15.3	2.2–48.8
depressive symptoms	18^e^	6.3 ± 6.4	5.0	0–38	6.6 ± 7.0	5.0	0–40

Note: *LPCBs* lower-chlorinated biphenyls (28, 52, 101), *HPCBs* higher-chlorinated biphenyls (138, 153, 180), *dlPCBs* dioxin-like polychlorinated biphenyls (105, 114, 118, 123, 156, 157, 167, 189), *HVA* homovanillic acid, *VMA* vanillylmandelic acid, *crea* creatinine, *SD* standard deviation, *t1* measurement occasion 1, *t2* measurement occasion 2

^a^in ng/g lipid

^b^in μmol/g Crea

^c^median of PCB exposure in the German general population; data source Schettgen et al. [23]

^d^intern laboratory reference value

^e^cut of value for clinically relevant depression [50]

### Statistical analyses

As the PCB sum variables were not normally distributed, we log-transformed them with a natural logarithm. Possible control variables (i.e. sex, age, education, work status, traumatic experience, co-exposure, total lipids and albumin) were analyzed with a directed acyclic graph. The online tool DAGitty version 2.3 was used to generate the graph [34]. The resulting graph is reported in Additional file 1: Figure S1. As minimal sufficient adjustment set three control variables were identified: 1) albumin as marker for liver function, 2) age as an influencing factor of PCB body burden and 3) traumatic experience as influencing factor for depressive symptoms as well as HVA level. Thus, we controlled for age and albumin in testing hypothesis 1 and we added traumatic experiences in all mediation analyses (hypothesis 2). The variable depressive symptoms at t1was included as further control variable in the longitudinal mediation analyses.

We analyzed the direct path between PCB body burden and depressive symptoms with multiple linear regression analyses to test the first hypothesis. PCB body burden was included as the independent variable, depressive symptoms as the dependent variable and age and albumin as control variables. The second hypothesis was tested with mediation analysis. The direct association of PCBs and depressive symptoms (hypothesis 1) is mediated by the levels of urinary DA metabolites HVA/crea and VMA/crea; specifically, PCB exposure is negatively associated with urinary DA metabolites. Reduced DA metabolites are in turn associated with more depressive symptoms. To test a mediation three steps have to be carried out [35]. Testing the direct path in hypothesis 1 is the first step (see Fig. 1). In the second step, the association between PCB body burden and the mediator (i.e. DA metabolites HVA/crea and VMA/crea) has to be tested. If both associations are significant, a mediation as the representing indirect path between the predictor and the outcome can be tested in the final step; both, PCB body burden and the DA metabolites are included in one model as independent variables. The direct path (i.e. the predictor variable) should lose its significant association with the outcome variable after including the mediator variable into the model. If the direct path is still significant, although the mediator variable has a significant effect on the outcome, it is called a partial mediation. The indirect path of PCB body burden and depressive symptoms through DA metabolites was tested with the MEDIATION macro for SPSS of Hayes [36]. At each measurement time point, depressive symptoms was used as the dependent variable, the respective PCB sum variables were included as independent variables and the metabolites HVA/crea and VMA/crea as mediators. The mediation analyses were performed to test cross-sectional mediations at t1 and t2 as well as to test longitudinal mediations with PCB exposure at t1 to depressive symptoms at t2.

All analyses were performed with IBM SPSS 21 for windows [37]. Due to the postulated directed hypotheses, the one-sided level of significance (*p* < .05) was used for the regression analyses. In the mediation tests, the 90%-confidence interval would be the equivalent for testing one-sided. Two analyses were performed for each hypothesis (cross-sectional and longitudinal) and thus a 95%-confidence interval was used to correct the tested indirect paths for multiple testing.

## Results

Two hypotheses were tested in this study. In the first hypothesis, the direct effect of PCB body burden on depressive symptoms was analyzed. We find significant positive associations between LPCBs and depressive symptoms as well as between dlPCBs and depressive symptoms for both measurement occasions (Table 2). The association between HPCBs and depressive symptoms is only significant at t2, but not at t1. Hypothesis one can be confirmed except for HPCBs at t1.

**Table 2:** Results of multiple linear regression analyses to test the direct path of PCB body burden and depressive symptoms (controlled for age and albumin)

IV	B	S.E.	β	t	p	R^2^
LPCBs_t1	0.81	.32	.28	2.56	.01	.07
HPCBs_t1	1.00	.55	.19	1.81	.07	.04
dlPCBs_t1	0.99	.44	.24	2.24	.03	.06
LPCBs_t2	0.76	.34	.23	2.21	.03	.16
HPCBs_t2	1.37	.56	.24	2.47	.02	.14
dlPCBs_t2	1.28	.44	.28	2.88	.005	.19

Notes: *PCBs* polychlorinated biphenyls, *LPCBs* lower-chlorinated PCBs, *HPCBs* higher-chlorinated PCBs, *dlPCBs* dioxin-like PCBs, *t1* measurement occasion 1, *t2* measurement occasion 2, *IV* independent variable, *B* unstandardized regression coefficient, *S.E*. standard error, *β* standardized regression coefficient, *t* t-value, *p p*-value (significance), *R* ^*2*^ explained variance

**Fig. 3:**
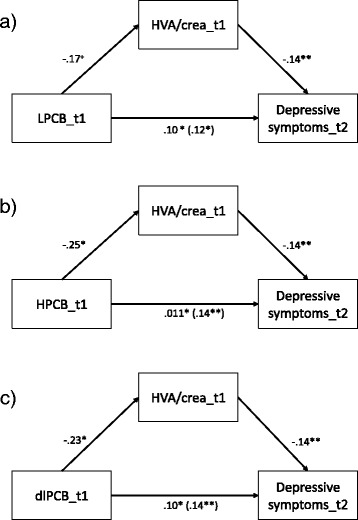
Illustration with standardized-b-coefficients of the longitudinal indirect paths of PCB to depressive symptoms through HVA/crea. Note: HPCB = higher-chlorinated PCBs, dioxin-like PCBs, HVA = homovanillic acid, t1 = measurement occasion 1, t2 measurement occasion 2; ^+^ = *p* < .10 (1-sided), * = *p* < .05 (1-sided)

In the second hypothesis, the indirect effect of PCB body burden on depressive symptoms mediated by DA metabolites HVA/crea and VMA/crea was investigated. There are no significant cross-sectional mediations for HVA/crea; neither for t1 nor for t2, but significant indirect paths over time are identified for all three PCB groups (i.e. LPCBs, HPCBs and dlPCBs; see Table 3). Specifically, HVA/crea at t1 partially mediates the association between PCB exposure at t1 and depressive symptoms at t2. After including the mediator, the direct path remains significant (see Fig. 3).

**Table 3:** B-coefficients for indirect path of PCB body burden to depressive symptoms trough dopamine metabolites HVA/crea and VMA/crea (bootstrapping with *N* = 5000)

Indirect path	Effect	S.E. (boot)	BC Bootstrapping 95% CI	
			Lower limit	Upper limit
Mediator HVA/crea				
LPCB_t1 ➔ HVA/crea_t1 ➔ depressive symptoms_t1	−.01	.04	−.10	.05
LPCB_t2➔ HVA/crea_t2 ➔ depressive symptoms_t2	−.001	.06	−.11	.13
LPCB_t1 ➔ HVA/crea_t1 ➔ depressive symptoms_t2^1^	*.10*	*.05*	*.02*	*.24*
LPCB_t1➔ HVA/crea_t2 ➔ depressive symptoms_t2	.06	.08	−.04	.31
HPCB _t1➔ HVA/crea_t1 ➔ depressive symptoms_t1	−.01	.07	−.21	.10
HPCB _t2➔ HVA/crea_t2 ➔ depressive symptoms_t2	.03	.08	−.09	.24
HPCB _t1➔ HVA/crea_t1 ➔ depressive symptoms_t2^1^	*.22*	*.10*	*.06*	*.50*
HPCB _t1➔ HVA/crea_t2 ➔ depressive symptoms_t2	.08	.13	−.10	.49
dlPCB_t1 ➔ HVA/crea_t1 ➔ depressive symptoms_t1	−.01	.05	−.15	.08
dlPCB_t2 ➔ HVA/crea_t2 ➔ depressive symptoms_t2	.02	.07	−.08	.20
dlPCB_t1 ➔ HVA/crea_t1 ➔ depressive symptoms_t2^1^	*.17*	*.08*	*.05*	*.36*
dlPCB_t1 ➔ HVA/crea_t2 ➔ depressive symptoms_t2	.08	.11	−.06	.41
Mediator VMA/crea				
LPCB_t1 ➔ VMA /crea_t1 ➔ depressive symptoms_t1	.00	.01	−.02	.05
LPCB_t2➔ VMA /crea_t2 ➔ depressive symptoms_t2	−.00	.02	−.05	.04
LPCB_t1 ➔ VMA /crea_t1 ➔ depressive symptoms_t2	.00	.05	−.06	.10
LPCB_t1➔ VMA /crea_t2 ➔ depressive symptoms_t2	−.00	.04	−.09	.08
HPCB _t1➔ VMA /crea_t1 ➔ depressive symptoms_t1	.00	.03	−.04	.08
HPCB _t2➔ VMA /crea_t2 ➔ depressive symptoms_t2	.00	.03	−.06	.08
HPCB _t1➔ VMA /crea_t1 ➔ depressive symptoms_t2	−.01	.09	−.08	.21
HPCB _t1➔ VMA /crea_t2 ➔ depressive symptoms_t2	.00	.07	−.15	.14
dlPCB_t1 ➔ VMA /crea_t1 ➔ depressive symptoms_t1	−.00	.02	−.59	.04
dlPCB_t2 ➔ VMA /crea_t2 ➔ depressive symptoms_t2	.00	.03	−.05	.07
dlPCB_t1 ➔ VMA /crea_t1 ➔ depressive symptoms_t2	.01	.08	−.07	.17
dlPCB_t1 ➔ VMA /crea_t2 ➔ depressive symptoms_t2	.00	.05	−.10	.12

Notes: *PCBs* polychlorinated biphenyls, *LPCBs* lower-chlorinated PCBs, *HPCBs* higher-chlorinated PCBs, *dlPCBs* dioxin-like PCBs, *HVA* homovanillic acid, *VMA* vanillylmandelic acid, *crea* creatinine, *t1* measurement occasion 1, *t2* measurement occasion 2, *S.E. (boot)* bootstrapped standard error, *BC* bias corrected, *CI* confidence interval; ^1^ significant mediations are in italic (*p* < .05)

Exposure with PCBs at t1 predicts depressive symptoms at t2. These direct effects are mediated by HVA/crea at t1. Detailed information regarding the analyses is reported in Tables 4, 5 and 6 and illustrations of the significant indirect paths with standardized beta coefficients are represented in Fig. 3. Thus, our longitudinal hypothesis can be partially confirmed for one indirect path via HVA/crea for all groups of PCBs (i.e. LPCBs, HPCBs and dlPCBs).

**Table 4:** Results of the mediation analyses of LPCB body burden and depressive symptoms through HVA/crea using MEDIATE macro for SPSS [30]; controlled for age, albumin and traumatic experience

	Coefficient	SE	t	p
Total effect model (DV = depressive symptoms_t2)				
LPCBs	.48	.24	2.05	.02
Covariates				
Age	−.05	.04	−1.17	.12
Albumin	−.12	.19	−0.60	.28
Traumatic experience	1.62	1.37	1.18	.12
Depressive symptoms_t1	.75	.09	8.60	<.001
Model fit				
R^2^	.62			<.001
F	26.67			
Effect of IV on HVA (mediator)				
LPCBs	−.75	.50	−1.50	.07
Effect of IV and mediator on depressive symptoms_t2				
HVA/crea	−.13	.05	−2.61	.01
LPCBs	.38	.23	1.67	<.05
Model fit				
R^2^	.65			<.001
F	24.92			
Homogeneity of regression (LPCB*HVA)				
R^2^	.0004			.38
F	0.10			

Note: *DV* dependent variable, *IV* independent variable, *t1* measurement occasion 1, *t2* measurement occasion 2, *LPCBs* lower-chlorinated biphenyls, *HVA* homovanillic acid / creatinine, *R* ^*2*^ explained variance, *F* F-value, *SE* standard error, *t* t-value, *p* p-value (significance)

**Table 5:** Results of the mediation analyses of HPCB body burden and depressive symptoms through HVA/crea using MEDIATE macro for SPSS [30]; controlled for age, albumin and traumatic experience

	Coefficient	SE	t	*p*
Total effect model (DV = depressive symptoms_t2)				
HPCBs	.94	.39	2.40	.01
Covariates				
Age	−.08	.04	−1.96	.03
Albumin	−.14	.19	−.72	.27
Traumatic experience	1.61	1.36	1.19	.12
Depressive symptoms_t1	.75	.09	8.79	<.001
Model fit				
R^2^	.62			<.001
F	27.45			
Effect of IV on HVA (mediator)				
HPCBs	−1.84	.82	−2.24	.02
Effect of IV and mediator on depressive symptoms_t2				
HVA/crea	−.12	.05	−2.40	.01
HPCBs	.71	.39	1.82	.04
Model fit				
R^2^	.65			<.001
F	25.17			
Homogeneity of regression (HPCB*HVA)				
R^2^	.002			.25
F	0.47			

Note: *DV* dependent variable, *IV* independent variable, *t1* measurement occasion 1, *t2* measurement occasion 2, *HPCBs* higher-chlorinated biphenyls, *HVA* homovanillic acid/creatinine, *R* ^*2*^ explained variance, *F* F-value, *SE* standard error, *t* t-value, *p* p-value (significance)

**Table 6:** Results of the mediation analyses of dlPCB body burden and depressive symptoms through HVA/crea using MEDIATE macro for SPSS [30]; controlled for age, albumin and traumatic experience

	Coefficient	SE	t	*p*
Total effect model (DV = depressive symptoms_t2)				
dlPCB	.80	.31	2.56	.02
Covariates				
Age	−.06	.04	−1.56	.07
Albumin	−.14	.19	−.72	.24
Traumatic experience	1.75	1.36	1.29	.10
Depressive symptoms_t1	.74	.09	8.62	<.001
Model fit				
R^2^	.63			<.001
F	27.86			
Effect of IV on HVA (mediator)				
dlPCB	−1.40	.67	−2.10	.02
Effect of IV and mediator on depressive symptoms_t2				
HVA/crea	−.12	.05	−2.41	.01
dlPCB	.63	.31	2.03	.03
Model fit				
R^2^	.65			<.001
F	25.53			
Homogeneity of regression (HPCB*HVA)				
R^2^	.002			.27
F	0.39			

Note: *DV* dependent variable, *IV* independent variable, *t1* measurement occasion 1, *t2* measurement occasion 2, *dlPCBs* dioxine-like polychlorinated biphenyls; HVA = homovanillic acid / creatinine, *R* ^*2*^ explained variance, *F* F-value, *SE* standard error, *t* t-value, *p* p-value (significance)

For VMA/crea neither the cross-sectional nor the longitudinal indirect paths are significant. Thus, for VMA/crea the second hypothesis is not supported.

## Discussion

Occupational and environmental exposures to PCBs have been associated with reduced well-being and depressive symptomatology [9]. In the present study, we investigated whether this effect is mediated through the urinary DA metabolites HVA and VMA. The mediations were tested cross-sectionally and longitudinally; cross-sectional results were non-significant. However, longitudinally LPCBs, HPCBs and dlPCBs showed a negative association with HVA at t1 and these reduced HVA levels were in turn associated with an increase in depressive symptoms at t2. An increase in LPCBs of 10 ng/g lipids results in one additional depressive symptom (of the 21 queried symptoms) through the mediator HVA. Respectively, an increase of approximately 5 ng/g lipids in HPCBs or in dlPCBs also results in one additional depressive symptom via the HVA as the mediator. These results indicate that PCB-related depressive symptomatology may be related to lower levels of central DA as they are reflected in lower levels of HVA.

The longitudinal mediation of PCB exposure via HVA/crea on depressive symptoms is in line with prior studies. Seegal et al. [38] exposed apes to different PCBs over 20 weeks and reported reduced DA concentrations in the striatum directly after the exposure. A system within the basal ganglia, the striatum, subserves motor, cognitive and limbic functions such as drive, learning, memory, emotion and vegetative regulation. Whilst there was a correlation between PCB exposure and the DA concentration directly after exposure, no correlation was found 24 and 44 weeks later, despite the DA concentration still being reduced. One explanation for these findings might be that PCBs cause irreversible damage to brain structures such as the death of dopaminergic neurons [38]. In fact, we also found a significant mediation only over time (after 56 weeks), but not cross-sectionally. Cell death of dopaminergic neurons can be a consequence of blocked vesicular monoamine transporter 2 (VMAT2). Richardson & Miller [39] report that an exposure with PCBs can cause lower reactivity of VMAT2. The authors suggested that DA cannot be transported in the vesicle and will be anaerobically metabolized in the presynapsis. Reactive oxygen species (ROS) will then be produced destroying the neuron and leading to cell death [40]. Finally, a reduced frequency of dopaminergic neurons will potentially lead to a reduced DA concentration in the striatum and cause depressive symptoms [13]. These findings may explain why we found mediations only occurring over time.

We did not identify any mediation of PCBs through VMA on depressive symptoms. A reason for this may be the metabolite itself. Primarily, HVA is the main metabolite of DA but VMA also of norepinephrine (NE). Although NE is transformed from DA, the results related to VMA may also be influenced by NE and its function as a stress hormone. NE is increased in moments of acute stress [41]. Paris et al. [42] report, that a thoracic computed tomography (CT) is associated with more psychological distress in asbestos exposed participants, who are aware of asbestos-related health risks. Thus, it can be argued that the examination itself triggers the perception of health risks related to PCB exposure in our cohort and this may be a source of stress for participants, and may therefore influence acute NE levels. This could make it difficult to identify an indirect effect via VMA. Furthermore, in our study we are not able to differentiate the separate effects of DA and NE related to VMA. Jimerson et al. [15] report a six times stronger reduced CSF-HVA level than CSF-VMA level in depressed participants. Thus, it is possible that our study population is too small to detect the weaker effect in VMA.

One strength of our study is that the found effects are free from a common-method bias typically discussed in psychological and social science research. Effects may be overestimated, due to the use of the same method to assess predictor and outcome variables. In the current study, different methods were used to assess predictor, mediator and outcome variable. PCBs were measured in blood plasma, DA metabolites in urine and depressive symptoms with a standardized screening questionnaire. Thus, a common-method-bias that overestimates our results can be excluded.

A systematic drop-out of study participants could also bias our results. There were many drop-outs in our study population, but analyses show, that there are no systematic differences in the relevant variables used in the current study. A strength of this study is the one year time lag between the measurement occasions. Depressive symptoms may be related to autumn and winter season [43]. Using yearly time intervals can reduce a possible bias due to seasonal influences on depressive symptomatology.

The longitudinally indirect effects may be weak. However, it needs to be considered that also many control variables were included. In the longitudinal analyses it was controlled for age, Albumin, traumatic experience and depressive symptoms at t1. Including depressive symptoms at t1 was necessary for the identification of the longitudinal indirect effect on depressive symptoms at t2, but this variable can explain much of the variance of depressive symptoms at t2. Therefore, it is a strength of the present study that we found significant mediations although we controlled for depressive symptoms at t1. This allows interpreting the results regarding the levels of DA metabolite HVA at t1 as an induced change in depressive symptoms at t2.

In epidemiologic research about hazardous substance exposure, it is necessary to discuss possible co-exposure to other substances. PCBs may be not the only hazardous substance that our study population is exposed to. The WHO TEQ for dioxins and furans was analyzed in our study cohort. However a co-exposure to other toxins does not produce an open backdoor-path in the DAG (see Additional file 1: Figure S1), because there is no association of dioxins and furans with depression; thus it is not part of the minimal adjustment set.

It is also important to note that the urinary metabolites may have originated in physiological structures other than the brain. For instance, only 12% of the urinary HVA originates from central DA [44]. Further, 94% of the urinary VMA originate from the liver [29] and PCBs are suspected to influence hepatic function [3]. The different sources of HVA and VMA may reduce the capacity of the study to detect a stronger effect on the outcome variable. Nevertheless, the use of urinary metabolites is a non-invasive method and we controlled for albumin as marker for liver function and also liver dysfunction in all analyses. Thus, abnormalities in liver function cannot explain the indirect effect of HVA related to depressive symptoms. Albumin is also a possible marker of longitudinal alcohol consumption, but by including albumin as control variable, we also controlled for longitudinal alcohol consumption. Furthermore, it has been reported that the measurement of HVA in blood correlates with renal clearance. Lambert et al. [45] conclude that blood concentration of HVA does not necessarily reflect the metabolism of DA but also depends on the amount of renal clearance. By measuring the urinary HVA we are able to control for renal clearance and urine density by adjusting for creatinine.

Finally, a controversial discussion in the literature focuses on the “dopamine hypothesis”, which states that low DA concentration may also cause depressive symptoms. It has been argued that DA is only relevant in certain forms of depressive symptoms (i.e. motor retardation [13]). Nevertheless, in our data there were no specific symptoms responsible for the association. This was true of symptoms related to motor activity as well as other symptoms measured (post hoc tests not shown in tables).

When interpreting the present findings, it is important to keep in mind that our study sample had a higher exposure to PCBs compared to other study samples where the impact of PCB exposure on mental health has been investigated (see Additional file 1: Table S1). This might explain why we were able to detect such small effects. Our work provides the first insight into a potential mechanism by which high-level PCB exposure influences the DA neurotransmitter system and increases symptoms over time. Yet, the pathophysiology of depression involves a complex interaction of neurotransmitters, hormones or behavioral factors. In order to address this complexity, future research should assess serotonin, which also plays a role in the development of depressive symptoms according to drug studies [46]. Additionally, the impact of other organ systems such as the thyroid gland should be considered, since PCBs have been argued to disturb thyroid hormones [47] and hypothyroidism is associated with depressive symptoms [48]. Finally, psychological mechanisms should also be considered when investigating the development of depressive symptoms after hazardous substance exposure. In a review, it was reported that patients show more depressive symptoms short time after getting the information of higher risk for developing a chronic disease [49]. A hazardous substance exposure might be threatening, because individuals are aware of the increased risk for developing a severe illness (i.e. cancer). Thus, this knowledge might cause a perceived health threat and may also play a role in developing mental syndromes [7, 9].

## Conclusion

We were able to support prior findings that a high PCB body burden is associated with more depressive symptoms. Furthermore, this study gives first hints that the relationship between all types of PCB exposure (LPCB, HPCB and dlPCB) with depressive symptoms may be mediated by alterations in DA metabolism over time. This study encourages further research to investigate the impact of PCBs on other monoamine neurotransmitters, in order to learn more about underlying pathomechanisms of PCBs and mental health.

## Additional file

Additional file 1: Figure S1. Directed acyclic graph to identify relevant control variables. **Table S1.** Mean PCB exposure of our study cohort in comparison to other study cohorts; sorted by PCB exposure (DOCX 89 kb)

## Abbreviations

BDI-II: Beck’s depression inventory II (German version)
CDC: Centers for disease control and prevention
Crea: Creatinine
CSF: Cerebrospinal fluid
DA: Dopamine
DAT: Dopamine transporter
dlPCBs: Dioxin-like polychlorinated biphenyls
DSM-IV: Diagnostic and Statistical Manual of Mental Disorders IV
HELPcB: Health effects in high level exposure to PCB
HPCBs: Higher chlorinated biphenyls
HPLC: High performance liquid chromatography
HVA: Homovanillic acid
LOD: Limit of detection
LPCBs: Lower chlorinated biphenyls
NE: Norepinephrine
PCBs: Polychlorinated biphenyls
VMA: Vanillylmandelic acid
