# Supplementary material for: Polychlorinated biphenyls and depression: cross-sectional and longitudinal investigation of a dopamine-related Neurochemical path in the German HELPcB surveillance program
Source: Environ Health. 2017 Oct 10;16:106. doi: 10.1186/s12940-017-0316-3 (PMC5635510; doi:10.1186/s12940-017-0316-3)
Supplement: Additional file 1: Figure S1. — Directed acyclic graph to identify relevant control variables. Table S1. Mean PCB exposure of our study cohort in comparison to other study cohorts; sorted by PCB exposure (DOCX 89 kb) [file 12940_2017_316_MOESM1_ESM.docx]

***Additional file***

**Polychlorinated Biphenyls and Depression: cross-sectional and longitudinal Investigation of a dopamine-related Neurochemical Path in the German HELPcB Surveillance Program**

**Petra Maria Gaum*, Monika Gube, Thomas Schettgen, Franziska Maria Putschögl, Thomas Kraus, Bruno Fimm, Jessica Lang**

*** Correspondence regarding this article should be sent to:**

Dr. Petra M. Gaum

Institute for Occupational and Social Medicine

University Hospital RWTH Aachen

Pauwelsstraße 30

52074 Aachen, Germany

Tel.: +49 241 80 89040

Fax: +49 241 80 82587

Email: pgaum@ukaachen.de

Web: www.arbeitsmedizin.ukaachen.de

Figure S1: Directed acyclic graph to identify relevant control variables


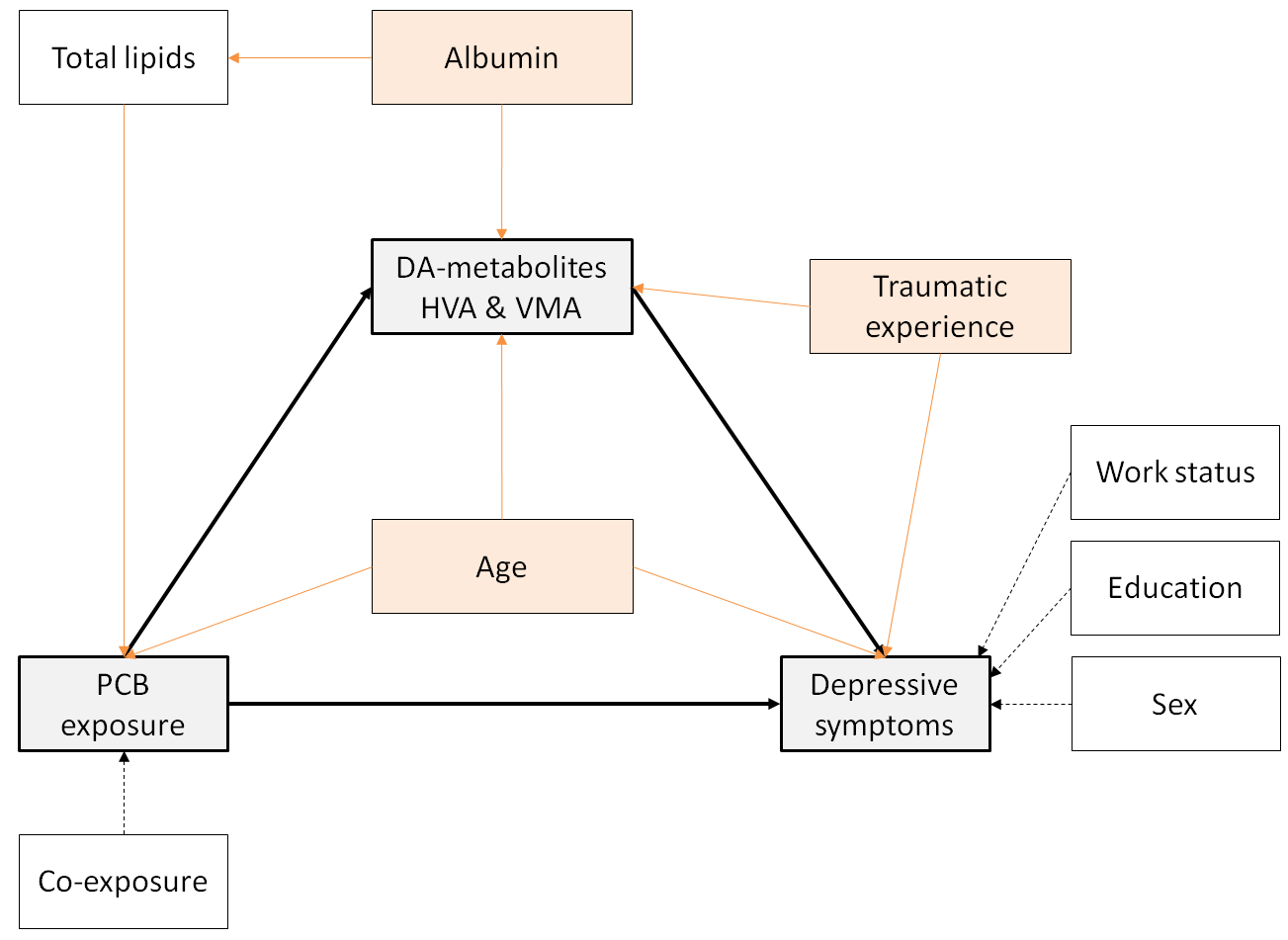


Notes: grey fields = mediation model; white fields = possible confounding variables; orange fields = minimal sufficient adjustment set

Table S1: Mean PCB exposure of our study cohort in comparison to other study cohorts; sorted by PCB exposure.

| Reference | Area | Type of exposure | Investigated PCBs | Mean serum PCB  (ng/g lipid) | No. of participants | Mean age | Effects |
| --- | --- | --- | --- | --- | --- | --- | --- |
| Current study t1 | Western Germany | occupational | ∑PCBs (14 congeners) | 1712,1 ng/g lipid | 155 men +  23 women | 46.9y | HVA ↓ 🡪 Depressive symptoms ↑  (mediation over time) |
| Current study t2 | Western Germany | occupational | ∑PCBs (14 congeners) | 1518,3 ng/g lipid | 155 men +  23 women | 47.9y |  |
| Seegal et al. [1] | New York State (USA) | occupational | ∑PCBs (23 congeners) | 1025 ng/g lipid² | 123 men +  107 women | men 63.9y  women 65.2y | State anxiety ↑ (moderate effect)  No effects for depression |
| Seegal et al. [2] | New York State (USA) | occupational | ∑PCBs (23 congeners) | 980 ng/g lipid ²  (geometric mean) | 50 men +  39 women | men 62.6y  women 64.6y | Striatal DAT density ↓ |
| Peper, Klett, Morgenstern [3] | South-western Germany | Indoor air | ∑PCBs (5 congeners) | 635.7ng/g lipid^1^ EG  521.4 ng/g lipid^1^ CG | EG 30 (60 % men)  CG 30 (40% men) | EG 48.2y  CG 49.9y | Psychological well-being ↓ (moderate effect) |
| Fitzgerald et al. [4] | New York State (USA) | environmental | ∑PCBs (30 congeners) | 536.8 ng/g lipid | 127 men +  126 women | 63.9y | Depressive symptoms ↑ |
| Lin et al. [5] | Taiwan (Yucheng) | nutritional | Kanechlor 500 | EG 78.2 ng/g lipid^3^  CG 50.1 ng/g lipid^3^ | EG 162 (50% men)  CG 151 (52% men) | EG 68.9y  CG 69.7y | No effects on depressive symptomatology |
| Santiago-Rivera et al. [6] | Akwasasne Mohawk reservation (upstate New York) | environmental | ∑PCBs (91 congeners) | 3.5 ng/g lipid | 113 men +  240 women | 38y | No effects on HVA and depressive symptoms |

Notes: EG =exposed group; CG = control group.

^1^ transformed in ng/g lipid serum by the authors

² mean calculated by the authors

^3^ initial PCB exposure 1980-1982

References:

1. Seegal RF, Fitzgerald EF, McCaffrey RJ, Shrestha S, Hills EA, Wolff MS, et al. Tibial bone lead, but not serum polychlorinated biphenyl, concentrations are associated with neurocognitive deficits in former capacitor workers. J Occup Environ Med. 2013; doi:10.1097/JOM.0b013e318285f3fd.
2. Seegal RF, Marek KL, Seibyl JP, Jennings DL, Molho ES, Higgins DS, et al. 2010. Occupational exposure to PCBs reduces striatal dopamine transporter densities only in women: A β-CIT imaging study. Neurobiol Dis. 2010; doi:10.1016/j.nbd.2010.01.009.
3. Peper M, Klett M, Morgenstern R. Neuropsychological effects of chronic low-dose exposure to polychlorinated biphenyls (PCBs): A cross-sectional study. Environ Health. 2005; doi:10.1186/1476-069X-4-22.
4. Fitzgerald EF, Belanger EE, Gomez MI, Cayo M, McCaffrey RJ, Seegal RF, et al. Polychlorinated biphenyl exposure and neuropsychological status among older residents of upper hudson river communities. Environ Health Persp. 2008; doi:10.1289/ehp.10432.
5. Lin LC, Guo NW, Tsai PC, Yang CY, Guo YLL. Neurocognitive changes among elderly exposed PCBs/PCDFs in Taiwan. Environ Health Perspect. 2008; doi: 10.1289/ehp.10134.
6. Santiago-Rivera A, Morse GS, Haase RF, McCaffrey, Tarbell A. Exposure to an environmental toxin, quality of life and psychological distress. J Environ Psychol. 2007; doi: 10.1016/j.jenvp.2006.12.004.
